# Incidence rate of post-intensive care syndrome-family in Japan: A post-hoc analysis of a prospective observational study

**DOI:** 10.2478/jccm-2025-0042

**Published:** 2025-10-31

**Authors:** Akihiro Takaba, Masaaki Sakuraya, Daisuke Kawakami, Shigeki Fujitani

**Affiliations:** JA Hiroshima Koseiren Hiroshima General Hospital, Hiroshima, Japan; Iizuka Hospital, Iizuka, Japan; St Marianna University School of Medicine, Kawasaki, Japan

**Keywords:** post-intensive care syndrome, post-intensive care syndrome-family, caregiver stress, caregiver burden, ICU liberation bundle

## Abstract

**Background:**

Family members in intensive care units (ICUs) may develop post-intensive care syndrome-family (PICSF), characterized by psychiatric disorders such as anxiety, depression, and post-traumatic stress disorders (PTSD). A previous study reported that approximately 13% of patient families in Japan develop PICS-F symptoms six months following ICU discharge, which is lower compared to other countries. However, this figure may be underestimated by administrative claims data in Japan. Although clinical guidelines recommend interventions to prevent PICS-F, the implementation rate of these interventions in Japan remains unclear. This study addresses the epidemiology of PICSF among family members of ICU survivors and the implementation of interventions for preventing PICS-F in Japan.

**Methods:**

A post-hoc analysis of a prospective multicenter cohort study was conducted, focusing on mechanically ventilated ICU survivors and their closest relatives. This study covered 16 ICUs in 14 hospitals between April 2019 and September 2020, using questionnaires to assess the PICS-F symptoms among relatives using the Hospital Anxiety (HADS-A) and Depression (HADS-D) Scale and the Impact of Event Scale-Revised (IES-R). The implementation rate of interventions to prevent PICS-F was also evaluated.

**Results:**

Of the 151 surveyed relatives, 104 relatives were assessed after 6 months. Notably, PICS-F was identified among 45.2% of relatives, with depression (36.5%), anxiety (31.7%), and PTSD (24.0%). Relatives with PICS-F were less likely to maintain their original employment compared to those without (61.3% vs 85.3%, P=0.047). While 63.5% of relatives received at least one preventive intervention during the ICU stay, more than one-third received none.

**Conclusions:**

The incidence of PICS-F in Japan is higher than previously reported, affecting nearly half of patient relatives. Moreover, the implementation rate of interventions to prevent PICS-F is low. These findings suggest the need for reinforced socioeconomic support.

## Background

Critically ill patients may experience residual deficits in their physical and cognitive function and mental health after discharge from intensive care units (ICUs), known as post-intensive care syndrome (PICS) [1, 2]. Furthermore, family members of these patients may also experience psychiatric disorders including anxiety disorders, depression, post-traumatic stress disorders (PTSD), and complicated grief. These disorders, termed PICS-family (PICS-F), may persist for a long time, even after discharge from an ICU [3]. Family members of ICU survivors often experience serious economic stress owing to changes in their employment or caregiving responsibilities [4, 5]. These burdens can have a long-lasting impact on their well-being and worsen the PICS-F symptoms.

Several studies have shown that more than one-third to half of family members continue experiencing some PICS-F symptoms [6, 7, 8, 9, 10, 11, 12]. A Japanese retrospective matched-pair cohort study found that 12.8% of spouses of ICU patients visited hospitals due to mental disorders within six months [13]. This finding may be underestimated as the previous result was based on an administrative claims database rather than a questionnaire [13]. Thus, the epidemiology of PICS-F among patient families in Japan remains unclear.

Several interventions to promote family engagement have been suggested in the ICU Liberation Bundle (Bundle F) to prevent PICS-F [14, 15]. Previous studies have demonstrated that flexible visitation policies and the involvement of family members in patient care were effective in reducing the symptoms of PICS-F [16, 17]. However, ICU diaries were not associated with a decrease in the symptoms [18]. Thus, the efficacy of all interventions included in Bundle F remains unestablished, despite the recommendation of guidelines for family-centered care in the ICU. The value of medical care differs according to race and ethnicity [19, 20]. Revealing the implementation of Bundle F in Japan is warranted for further interventional studies.

We conducted a post-hoc analysis of the Japanese PICS (J-PICS) study, a prospective multicenter cohort research of mechanically ventilated ICU patients [21]. This post-hoc analysis aimed to address the epidemiology of PICS-F among ICU survivors’ family members and the implementation rate of interventions for preventing PICS-F in Japan.

## Methods

### Study design and setting

This study is a post-hoc analysis of the J-PICS study that was conducted in 16 ICUs across 14 hospitals in Japan between April 01, 2019 and September 30, 2019 [21]. Overall, 5 of the 16 ICUs were university-affiliated hospitals, while the others were tertiary teaching ones. This post-hoc analysis was approved by the institutional review board of JA Hiroshima General Hospital (approval number: 24–6) and reported according to the Strengthening the Reporting of Observational Studies in Epidemiology Statement.

### J-PICS study

Briefly, the J-PICS study enrolled critically ill adult patients who required mechanical ventilation for > 48 hours. Patients with the following conditions were excluded: primary brain injuries that could lead to conscious or cognitive disorders, a pre-admission diagnosis of dementia, home ventilation prior to admission, end-stage cancer, withdrawing/withholding status, and those expected to pass away within 24 hours. Six months after the ICU admission, questionnaires were mailed to families to survey the symptoms and life-styles of all patients and relatives, except those already known to have died. The study protocol was approved by the ethics committees of the Kobe City Medical Center General Hospital and all participating hospitals (approval number: Zn181008). Written informed consent was obtained from all patients or authorized surrogates. The J-PICS study was registered with the University Hospital Medical Information Network Clinical Trials Registry on October 26, 2018 (registration number: UMIN000034072).

### Participants

This post-hoc analysis used data from the J-PICS data-set, which includes information on both patients and their closest relatives. The closest relatives of the ICU survivors answered questions about the symptoms of PICS-F and lifestyle. From the J-PICS dataset, this post-hoc analysis included relatives who responded to questions and completed at least the Hospital Anxiety and Depression Scale (HADS) [22, 23] and Impact of Event Scale-Revised (IES-R) [24, 25]. The HADS was used for measuring anxiety (HADS-A) and depression (HADS-D) and the IES-R for measuring PTSD.

### Variables and measurements

This study primarily focused on the psychological outcomes of family members. Relatives’ baseline information included age, sex, and relationship with the patient. To provide clinical context, we also collected patient information during ICU admission, which included age, sex, clinical frailty scale score, acute physiology and chronic health evaluation score, sequential organ failure assessment (SOFA) score, duration of mechanical ventilation, ICU and hospital length of stay, and discharge destination. At 6 months after ICU admission, PICS was evaluated using questionnaires based on the 36-item Short Form (SF-36) [26, 27] and the Short-Memory Questionnaire (SMQ) [28, 29]. It was defined by the following criteria: (1) decline in physical status, indicated by a physical component score decrease of ≥ 10 points (SF-36); (2) deterioration in mental status, demonstrated by a mental component score decrease of ≥ 10 points (SF-36); or (3) cognitive function impairment, indicated by a decline in the SMQ score and an SMQ score of < 40 at 6 months after the ICU admission.

### Family member-reported outcomes six months after the ICU admission

We evaluated the incidence of PICS-F among the closest relatives of the ICU survivors. PICS-F is defined as the presence of anxiety, depression, or PTSD, determined by HADS-A ≥ 8, HADS-D ≥ 8, and IES-R ≥ 25, respectively. To explore its risk factors, we compared relatives who developed PICS-F (PICS-F group) with those who did not (non-PICS-F group).

### Implementation of the Bundle F Interventions

We assessed the implementation of Bundle F, which included five interventions: (1) A family conference that involved structured discussions with healthcare providers within the first three days of ICU admission, based on the VALUE approach, a structured communication framework designed to Value family input, Acknowledge emotions, Listen, Understand the patient as a person, and Elicit questions [30]. (2) Flexible visitation allowed the family access beyond usual time or age restrictions. (3) Participation in interdisciplinary rounds implied that families joined team discussions regarding patient care. (4) Bedside care involvement referred to direct participation in the patient’s care or rehabilitation. (5) ICU diaries were written logs shared with families to support communication and memory during critical illness. Table 1 provides the operational definitions are provided.

**Table 1.** Definitions of the interventions included in Bundle F.

**Interventions**	**Definitions**
Family conference	A structured meeting held by the third day of ICU admission using the VALUE approach (Valuing family input, Acknowledging emotions, Listening, Understanding the patient as a person, and Eliciting questions) [30].
Flexible visitation	Family members were permitted to visit with relaxed restrictions regarding time of day and visitor’s age.
Involvement of family in interdisciplinary rounds	Family members were invited to participate in the multidisciplinary team rounds during the patient’s ICU stay.
Family participation in bedside care	Family members were engaged in bedside care or rehabilitation activities during the patient’s ICU stay.
ICU diary	A written journal was maintained to record the patient’s ICU course and facilitate communication with family members.

Implementation was defined as at least one occurrence during ICU stay, except for family conferences which required implementation within the first three days of admission.

### Statistical analysis

The data were expressed as numbers with corresponding percentages for dichotomous variables and as medians with interquartile ranges (IQR). In all analyses, the number of cases with missing data was reported; further, these cases were excluded from each analysis. Univariate analyses were performed using the Wilcox-on Rank Sum and Fisher’s Exact Tests for continuous and binary variables, respectively, to compare the PICSF and non-PICS-F groups. All statistical tests were two-sided, with a statistical significance of p-value < 0.05. We also assessed the implementation of various interventions included in Bundle F according to the type of facility (university-affiliated and tertiary teaching hospitals). Statistical analyses were performed using Stata 15.1 (StataCorp LLC, College Station, TX, USA).

## Results

**Fig. 1.**
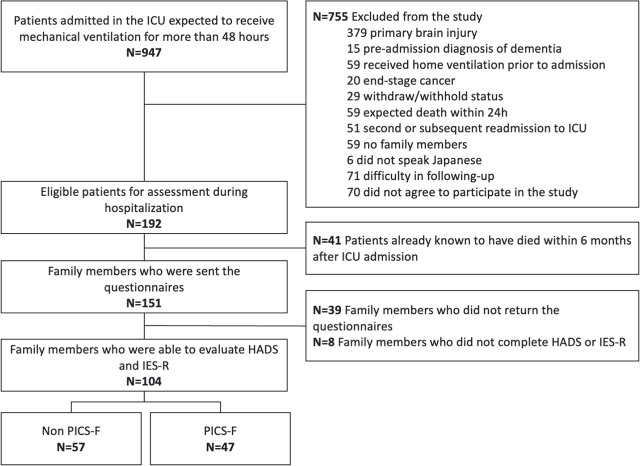
**Flow diagram of patient relatives.** Abbreviation: HADS: Hospital Anxiety and Depression scale; ICU: intensive care unit; IES-R: Impact of Event Scale-Revised; PICS-F: post-intensive care syndrome family.

In total, 192 patients were included in the J-PICS study. After excluding 41 patients who died within 6 months of follow-up, we sent questionnaires to 151 family members. Of these, 39 family members did not return the questionnaire survey, and 8 did not complete the HADS or IES-R questionnaires. Thus, 104 relatives were assessed for the outcomes after 6 months (Figure 1).

### Characteristics of the patients

The demographic and clinical characteristics of ICU patients are summarized in Table 2. The median patient age was 74 years (range, 61–81 years), and 67.3% were male. The median duration of mechanical ventilation, ICU stay, and hospital stay were 5 (range, 4–11), 8 (range, 5–14), and 37 (range, 21–67) days, respectively. Nearly half of the patients were discharged (47.1%). The SOFA score was slightly higher (8 [range, 6–11] vs. 7 [ 5, 6, 7, 8,9]), and fewer patients were discharged and sent home (17 [36.2%] vs. 32 [56.1%]) in the PICS-F group than in the non-PICS-F one.

**Table 2.** Characteristics of patients and patient relatives

	**Overall (N=104)**	**Non-PICS-F (N=57)**	**PICS-F (N=47)**	**P value**
Characteristics of patients				
Age, years, median [IQR]	74 [61–81]	74 [66–81]	74 [53.5–81]	0.427
Female, N (%)	34 (32.7)	18 (31.6)	16 (34.0)	0.836
APACHE II score, median [IQR]	21 [17–25]	20 [16–25]	23 [18–26]	0.113
SOFA score, median [IQR]	8 [5–10]	7 [5–9]	8 [6–11]	0.045
Clinical frailty score, median [IQR]	3 [2–4]	3 [2–4]	3 [2–4]^a^	0.841
ICU length of stay, days, median [IQR]	8 [5–14]	8 [6–13]	7 [5–14.5]	0.906
Hospital length of stay, days, median [IQR]	37 [21–67]	35 [18–57]	45 [21.5–82.5]	0.213
Duration of mechanical ventilation, days, median [IQR]	5 [4–11]	5 [3–9]	5 [2–10]	0.755

Discharged from hospital among survivors				0.050
Another facility, N (%)	55 (52.9)	25 (43.9)	30 (63.8)	
Nursing home, N (%)	0 (0)	0 (0)	0 (0)	
Home, N (%)	49 (47.1)	32 (56.1)	17 (36.2)	
PICS, N (%)	57 (54.8)^d^	28 (49.1)^b^	29 (61.7)^c^	0.197

Characteristics of patient relatives				
Age, years, median [IQR]	66 [56–72]	66 [58–72]^e^	65 [51.5–74]^f^	0.637
Female, N (%)	76 (73.0)	44 (77.2)	32 (68.1)	0.375
No regular employment at study enrollment, N (%)	39 (37.5)	23 (40.4)	16 (34.0)	0.547

Relationship to patient				0.330
Spouse, N (%)	51 (49.0)	29 (50.9)	22 (46.8)	
Parent, N (%)	14 (13.5)	5 (8.8)	9 (19.1)	
Child, N (%)	29 (27.9)	15 (26.3)	14 (29.8)	
Sibling, N (%)	6 (5.8)	5 (8.8)	1 (2.1)	
Others, N (%)	4 (3.8)	3 (5.3)	1 (2.1)	

a 1 missing data;

b 8 missing data,

c 6 missing data,

d 14 missing data,

e 12 missing data,

f 7 missing data; APACHE-II,

Acute Physiology and Chronic Health Evaluation; ICU, intensive care unit; IQR, interquartile ranges; PICS, post intensive care syndrome; PICS-F, post intensive care syndrome family; SOFA, Sequential Organ Failure Assessment.

### Characteristics of the relatives

Table 2 shows the characteristics of patients and their relatives. The median (IQR) relative age was 66 years (range, 56–72 years). Most of the relatives were female (73.0%), and almost half were patients’ spouses or partners (49.0%).

### Incidence of PICS-F symptoms

**Fig. 2.**
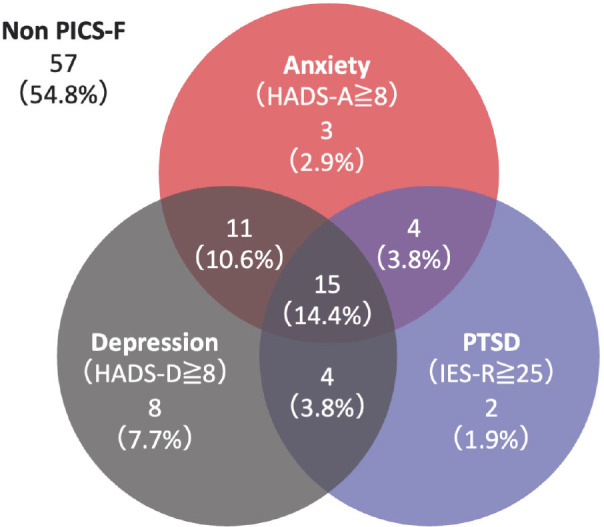
**Occurrence of PICS-F symptoms in 47 relatives who developed PICS-F.** Abbreviation: HADS: Hospital Anxiety and Depression scale; IES-R: Impact of Event Scale-Revised; PICSF: post-intensive care syndrome family.

Among 104 relatives assessed at 6 months, 47 (45.2%) met the criteria for PICS-F. Anxiety, depression, and PTSD symptoms were observed in 33, 38, and 35 relatives, respectively. Of the 47 relatives with PICS-F, 15 (31.9%), 19 (40.4%), and 13 (27.7%) had either all, two, or only one type of PICS-F symptom, respectively (Figure 2). The median HADS-A, HADS-D, and IESR scores were 9 (range, 7–13), 11 (range, 8–12), and 27 (range, 19–35), respectively, which were all over the thresholds for anxiety, depression, and PTSD (Table 3).

**Table 3.** Details for PICS-F symptoms and occupation status of patient relatives at six months after ICU admission

	**Overall (N=104)**	**Non-PICS-F (N=57)**	**PICS-F (N=47)**	**P value**
PICS-F symptoms				
HADS-A, median [IQR]	6 [3–9]	3 [2–5]	9 [7–13]	< 0.001
HADS-D, median [IQR]	5.5 [3–10]	4 [3–5]	11 [8–12]	< 0.001
IES-R, median [IQR]	12 [6–24]	6 [3–9]	27 [19–35]	< 0.001

Occupation status^a^				0.101
Continue in the same work, N (%)	48 (73.8)	29 (85.3)	19 (61.3)	0.047
Change in occupation status, N (%)	12 (18.5)	4 (11.8)	8 (25.8)	0.204
Leaving or losing the job, N (%)	5 (7.7)	1 (1.8)	4 (12.9)	0.184

a Excluded 39 patient families who was not employed at study enrollment. The HADS-A scores for anxiety and the HADS-D scores for depression. HADS, Hospital Anxiety and Depression scale; ICU, intensive care unit; IES-R, Impact of Event Scale-Revised; IQR, interquartile ranges; PICS-F, post intensive care syndrome family.

### Impact on occupational status

After excluding relatives who were unemployed at ICU admission, the proportion of relatives continuing in the same job was significantly lower in the PICS-F group (61.3% vs 85.3%, P=0.047). Changes in occupation status, including reduced working hours or retirement, were reported more frequently in the PICS-F group, although not significantly different.

### Implementation of Bundle F Interventions

**Fig. 3:**
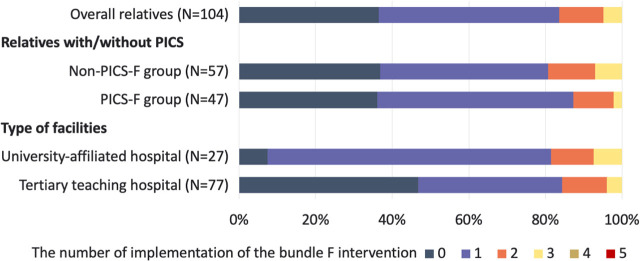
**Proportion of relatives who received Bundle F interventions.** Twenty-seven relatives received interventions in five university-affiliated hospitals, and 77 relatives in 11 tertiary teaching hospitals. Abbreviation: PICS-F: post-intensive care syndrome family.

Sixty-six (63.5%) relatives indicated receiving at least one of the interventions in Bundle F (Table 4). While one or two kinds of interventions were commonly performed, no relatives received more than four (Figure 3). The most frequently implemented intervention was a family conference within the first three days (33.3%). This was followed by family participation in bedside care (19 relatives, 18.3%) and flexible visitations (7 relatives, 6.7%). The implementation of most interventions included in Bundle F did not differ between the PICSF and non-PICS-F groups; however, ICU diaries were performed less frequently in the former (0% vs. 10.5%, P=0.031). When evaluated according to the facility type, more relatives received at least one intervention in the university hospital (92.6% vs. 53.2%, P< 0.001). The proportion of relatives who received one intervention was higher in the university hospital, whereas those who received two or more interventions were similar between the university and tertiary teaching hospitals.

**Table 4.** Implementation of bundle F during ICU stay

	**Overall (N=104)**	**Relatives with/without PICS**		**P value**	**Type of facilities**		**P value**
		**Non-PICS-F (N=57)**	**PICS-F (N=47)**		**University-affiliated hospital (N=27)**	**Tertiary teaching hospital (N=77)**	
Implementation of at least one of interventions, N (%)	66 (63.5)	36 (63.2)	30 (63.8)	1.00	25 (92.6)	41 (53.2)	< 0.001
Bundle components							
Family conference *, N (%)	103 (33.3)	49 (28.6)	54 (39.0)	0.290	37 (46.9)	66 (28.6)	0.0026
Flexible visitation, N (%)	7 (6.7)	4 (7.0)	3 (6.4)	1.00	3 (11.1)	4 (5.2)	0.372
Involvement of family in interdisciplinary rounds, N (%)	6 (5.8)	4 (7.0)	2 (4.3)	0.687	0 (0)	6(7.8)	0.335
Family participation in bedside care, N (%)	19 (18.3)	11 (19.3)	8 (17.0)	0.804	4 (14.8)	15 (19.5)	0.774
ICU diary, N (%)	6 (5.8)	6 (10.5)	0 (0)	0.031	2 (7.4)	4 (5.2)	0.648

* The number of conferences held within 3 days of ICU admission and that divided by patient-days are shown. ICU, intensive care unit; IQR, PICS-F, post intensive care syndrome family.

## Discussion

### Key findings

Of the 151 family members who were sent questionnaires, 104 (68.9%) closest relatives were assessed for outcomes after 6 months. Most relatives were female, and about half were spouses/partners. The incidence of PICS-F was 45.2% among the relatives of ICU survivors. Depression was the most common symptom observed in 36.5% of the relatives, followed by anxiety (31.7%) and PTSD (24.0%). Relatives with PICS-F reported a change in their occupational status at six months more frequently than those without PICS-F. Sixty-six relatives (63.5%) underwent one or more interventions in the ICU. However, more than one-third of the relatives received no intervention during their ICU stay.

### Association with previous studies

This study found a higher incidence of PICS-F (45.2%) among ICU survivors’ relatives as compared to a previous study using administrative claims data in Japan (12.8%) [13]. The latter study may have underestimated the incidence because only family members who received medical care for mental disorders were analyzed. Our study, using questionnaires with a relatively high response rate, revealed that the incidence of PICS-F was comparable to that of studies conducted in other countries, accounting for 35–50% [3,7]. According to the results of a systematic review, the prevalence of major symptoms of PICS-F, including depression (36.5%), PTSD (33.7%), and anxiety (31.7%), paralleled our findings [12]. Therefore, it is possible that some patient families with PICS-F did not receive medical care in Japan.

After the patients are discharged following recovery from a critical illness, caregiver burden and financial stress are common in patient families [31]; they are associated with symptoms such as anxiety and depression [4, 5,31]. In this study, the median ages of patients and their relatives were 74 and 66 years, respectively. Half of the relatives were spouses and most of them were female. After excluding unemployed relatives at ICU admission, fewer relatives with PICS-F remained in the same job as those without PICS-F. As unemployment is reported to be a potential risk factor for depression and anxiety in patient families [32], more socioeconomic support should be provided for caregivers to reduce their financial stress.

In our study, the proportion of relatives with one or more interventions included in Bundle F was 63.5%, similar to the results of an observational study involving 15,000 patients in the United States [33]. However, when checking the implementation rate of each intervention included in the bundle, most interventions were applied at a lower rate than in previous studies, except at family conferences. Family involvement in interdisciplinary rounds, bedside care, flexible visitation, and ICU diaries were implemented in 5.8%, 18.3%, 6.7%, and 5.8% of the patients, respectively. These implementation rates were lower than those reported in previous studies as 34% [33], 44% [33], 35% [34], and 17% [35], respectively. The feasibility of implementing such interventions appears to vary by country and influenced by local circumstances and cultural values. This suggests that involving families in care may be challenging in Japan. We also found differences in the implementation rate of family conferences between the university and tertiary teaching hospitals. Facility characteristics should be considered to promote the implementation of these interventions.

A flexible visitation policy has been associated with a reduction in anxiety and depression among family members [16]; moreover, family involvement in caregiving has been associated with fewer PTSD symptoms [17]. In addition, targeted support for the relatives of patients who died in ICUs may alleviate prolonged grief [36]. However, these findings are not universally consistent and highlight the need for further research in different cultural and religious contexts. Cultural, religious, and national differences influence medical preferences and values [20, 37]. Meanwhile, a lower implementation rate of Bundle F interventions may be attributable to insufficient evidence to guarantee the efficacy of the interventions suggested in the current guidelines advocating family-centered care in the ICU. In this study, the implementation rate of ICU diaries was higher in the non-PICS-F group. This finding suggested a potential association between the use of ICU diaries and lower incidence of PICS-F. However, the observed difference in use of ICU diaries, an intervention included in the Bundle F, might be a Type I error, as no significant differences were observed in the other components. Therefore, we were unable to draw conclusions regarding the usefulness of ICU diaries in the Japanese context. Further studies should explore the association between the implementation rate of the Bundle F interventions, including the ICU diaries, and incidence of PICS-F in Japan.

No prospective studies were performed to assess the incidence of PICS-F in Japan. The J-PICS study was conducted prospectively with a high response rate for questionnaires. In addition, our findings suggest that the epidemiology of PICS-F has been underestimated, but comparable to that in other countries. Meanwhile, Bundle F interventions were performed less frequently. Further studies are warranted to clarify interventions that are most cost-effective and feasible to implement in Japan. Individual approaches for each patient and relative may be better because of limited available data on the preferences and values of patients and family members, rather than adapting all Bundle F interventions.

### Study limitations

Our study has several limitations. First, critically ill adult patients who were expected to require mechanical ventilation for > 48 hours were enrolled. Although this study did not show the epidemiology of all ICU patients and family members, those with expected shorter ICU stays are likely to be at a lower risk of PICS-F. Second, the exclusion of family members of patients who died within six months may have introduced selection bias and led to an underestimation of the true burden of PICS-F. This limitation was due to the design of the original J-PICS study, which focused on assessing long-term outcomes among ICU survivors and their families. While this approach did not allow for the inclusion of bereaved families—who may also be at high risk for psychological symptoms [7, 8, 36]—it enabled a focused evaluation of psychological outcomes among relatives of surviving patients, a group that is also clinically important in the context of PICS-F. Third, although 68.9% of family members completed the questionnaire, non-respondents might have different mental health profiles, which could have led to potential selection bias. However, this response rate was comparable to that in previous PICS-F studies [8, 9]; furthermore, such levels of follow-up have been considered acceptable in longitudinal studies involving ICU families. Forth, the assessment of adherence to Bundle F interventions within the first three days may partially capture the entire implementation. It is difficult to compare with previous research because the bundle elements, definitions, and study durations for evaluating implementation differed across studies. This limitation underscores the need for standardized definitions and longer observation periods in future research. Finally, this study was conducted in Japan, and its generalizability is limited because the implementation of these interventions may vary by region and ethos. In addition, the use of virtual visits has grown since the coronavirus disease 2019 (COVID-19) pandemic [38]. This study was conducted before the onset of the COVID-19 pandemic and may differ from the current practices.

## Conclusion

Our study shows that the incidence of PICS-F was 45.2% in Japan, although this may be underestimated, it is comparable to that in other countries. The relatives of ICU survivors often face changes in their occupational status, indicating the need for further socioeconomic support. Although the effectiveness of Bundle F interventions remains unclear, its low implementation rate, except at family conferences, suggests cultural and institutional barriers to its application. Future research should focus on identifying specific barriers to intervention implementation and on developing culturally appropriate evidence-based support strategies that consider the diverse needs and circumstances of families.

## List of abbreviations

COVID-19: Coronavirus disease 2019
HADS: Hospital Anxiety and Depression Scale
HADS-A: Hospital Anxiety and Depression Scale -Anxiety Subscale
HADS-D: Hospital Anxiety and Depression Scale - Depression Subscale
ICU: Intensive care unit
IES-R: Impact of Event Scale-Revised
IQR: Interquartile ranges
J-PICS: Japanese post-intensive care syndrome study
PICS: Post-intensive care syndrome
PICS-F: Post-intensive care syndrome-family
PTSD: Post-traumatic stress disorder
SF-36: 36-item Short Form
SMQ: Short-Memory Questionnaire
SOFA: Sequential organ failure assessment
